# *GmCYP82A3*, a Soybean Cytochrome P450 Family Gene Involved in the Jasmonic Acid and Ethylene Signaling Pathway, Enhances Plant Resistance to Biotic and Abiotic Stresses

**DOI:** 10.1371/journal.pone.0162253

**Published:** 2016-09-02

**Authors:** Qiang Yan, Xiaoxia Cui, Shuai Lin, Shuping Gan, Han Xing, Daolong Dou

**Affiliations:** 1 Department of Plant Pathology, Nanjing Agricultural University, Nanjing, China; 2 National Center for Soybean Improvement, National Key Laboratory of Crop Genetics and Germplasm Enhancement, Nanjing Agricultural University, Nanjing, China; Chinese University of Hong Kong, HONG KONG

## Abstract

The cytochrome P450 monooxygenases (P450s) represent a large and important enzyme superfamily in plants. They catalyze numerous monooxygenation/hydroxylation reactions in biochemical pathways, P450s are involved in a variety of metabolic pathways and participate in the homeostasis of phytohormones. The *CYP82* family genes specifically reside in dicots and are usually induced by distinct environmental stresses. However, their functions are largely unknown, especially in soybean (*Glycine max* L.). Here, we report the function of *GmCYP82A3*, a gene from soybean *CYP82* family. Its expression was induced by *Phytophthora sojae* infection, salinity and drought stresses, and treatment with methyl jasmonate (MeJA) or ethephon (ETH). Its expression levels were consistently high in resistant cultivars. Transgenic *Nicotiana benthamiana* plants overexpressing *GmCYP82A3* exhibited strong resistance to *Botrytis cinerea* and *Phytophthora parasitica*, and enhanced tolerance to salinity and drought stresses. Furthermore, transgenic plants were less sensitive to jasmonic acid (JA), and the enhanced resistance was accompanied with increased expression of the JA/ET signaling pathway-related genes.

## Introduction

Soybean (*Glycine max* L.) is an agronomic crop grown throughout the world. It is not only an important source of vegetable protein and oil for humans and animals, but it is also a source of biofuels. In addition, soybean seeds contain a number of high-value secondary compounds with nutraceutical properties such as isoflavones, saponins, and tocopherols [1–3]. Phytophthora root and stem rot caused by *Phytophthora sojae* is one of the most destructive soybean diseases, results in annual losses of $200 million in the USA and $1–2 billion globally [4]. The effective way to protect soybean against *P*. *sojae* infection is breeding with dominant *Rps* (Resistance to *Phytophthora* s*ojae*) genes. But continuous utilization of a single *Rps* gene may result in selection pressure and promote the evolution of more pathogenic races of *P*. *sojae*. Thus, a particular *Rps* gene is effective for only 8 to 15 years. Partial resistance is another type of resistance which has been described as the relative ability of susceptible plants to survive infection without showing severe symptoms like death, stunting, or yield loss. It is conferred by multiple genes or quantitative trait loci (QTL), sometimes referred as quantitative, rate-reducing, or field resistance [5–7]. It is durable against all races of *P*. *sojae* and highly heritable [8, 9]. Therefore, partial resistance provides an effective way to develop Phytophthora resistant cultivars.

Till now, more than twenty QTL for partial resistance to *P*. *sojae* have been mapped in recombinant inbred line (RIL) populations. Most of the alleles of the QTL for partial resistance originating from Conrad, which is a proverbial cultivar with highly partial resistance to *P*. *sojae* [10–14]. At the same time, additional QTL were identified in V71-370 × PI 407162, Su88-M21 × Xinyixiaoheidou, S99-2281 × PI 408105A and OX20-8 × PI 398841 populations [15–19]. Identifying the key genes controlling these QTL and characterizing their functions will facilitate to understand the mechanisms that contribute to partial resistance. Generally, the genes that encode pathogenesis-related (PR) protein PR1a, PR2, basic peroxidase, and matrix metalloproteinase were present at higher abundances in partial resistant cultivars during infections [20]. The amount of preformed suberin was also found contribute to the partial resistance [21, 22]. Using soybean Aymetrix gene chips, whole-genome transcription profiles were analyzed in soybean genotypes with differential levels of partial resistance [8, 13, 23]. Appraising the differential transcript genes in Conrad and Sloan underlying the QTL found that most of them encompassed putative physiological trait genes, defense-related genes, and disease resistance-like genes [13, 14]. But the evidence of these candidate genes conferring resistance to *P*. *sojae* are still absent.

The cytochrome P450 (CYP) monooxygenases represent a large and important superfamily in plants. The enzymes catalyze a wide variety of monooxygenation/hydroxylation reactions in biochemical pathways involved in primary and secondary metabolism pathways [24, 25]. *P450* genes also participate in the homeostasis of phytohormones [25]. In soybean, the cytochrome *P450* gene family is comprised of 322 genes and 378 pseudogenes, but the biological functions most of them have not been elucidated [26]. The CYP82 family, which belongs to the CYP71 clan, is only present in dicots [27]. Some CYP82 members are reported to be highly induced by environmental stress in tobacco (*Nicotiana tabacum* L.), pea (*Pisum sativum* L.), soybean, and *Arabidopsis* [28–31]. A few CYP82 family members are involved in a variety of metabolic pathways. CYP82E4v1, from *N*. *tabacum*, was identified as a nicotine N-demethylase and the synthesis of nornicotine was suppressed in plants when the gene was silenced [32]. CYP82N2v2, from *Eschscholzia californica*, is involved in sanguinarine biosynthesis by catalyzing the conversion of protopine to dihydrosanguinarine by the P450 reaction [33]. CYP82D is reported to be involved in lipophilic flavone biosynthesis in sweet basil (*Ocimum bacilicum* L.) [34]. In *Arabidopsis*, CYP82G1 catalyzes the final step in the synthesis of the common plant homoterpene volatiles TMTT/DMNT, CYP82C2 and CYP82C4 hydroxylate the therapeutic compound 8-methoxypsoralen [35, 36]. The *CYP82* family genes also participate in the interaction between plants and pathogens. For example, cotton (*Gossypium hirsutum* L.) *CYP82D* can regulate systemic cell death by modulating the octadecanoid pathway and negatively regulate disease resistance to *Verticillium dahliae* by controlling JA biosynthesis [37]. *AtCYP82C2* can increase expressions of the JA-induced defense-related genes and contents of JA-induced IGs, and enhance resistance to *Botrytis cinerea* [38].

*GmCYP82A3* was highly stress responsive and located in the *P*. *sojae* resistant QTL region [13, 28]. But the accurate function remains unknown. In this study, we characterized the functions of *GmCYP82A3* in response to biotic and abiotic stresses. Ectopic expression of *GmCYP82A3* in *N*. *benthamiana* enhanced resistance to the pathogen *Botrytis cinerea* and *Phytophthora parasitica*, tolerance to the abiotic salinity and drought stresses. Furthermore, we demonstrated that JA/ET signaling pathway was altered in the *GmCYP82A3* overexpression plants. These findings provide valuable information on soybean partial resistance mechanisms.

## Materials and Methods

### Plant materials and growth conditions

The soybean cultivars Sloan (highly susceptible to *P*. *sojae*), Williams (moderately partial resistance to *P*. *sojae*) and Conrad (highly partial resistance) [8] were grown in a greenhouse. The greenhouse was maintained at 25°C and the photoperiod was set to 8D:16L. The *N*. *benthamiana* plants used were cultivated in the greenhouse under the same conditions.

### Gene induction assays

The germplasm Conrad was used for gene expression assay. The detached leaves from 3-week-old seedlings were used for *P*. *sojae* infection. Hyphae disks were cut from the edges of newly cultured *P*. *sojae* P6497 isolate on 10% V8 medium [39]. The inoculated leaves were placed in Petri dishes, which contained a layer of filter paper to retain moisture. Then the Petri dishes were placed in a climate chamber and samples were taken from the inoculated site at 0, 3, 6, 12, and 24 hours post-infection (hpi).

For salinity and drought stress treatments, three-week-old seedlings were uprooted and washed to remove vermiculite. The plants were cultured in Hoagland liquid medium [40] for 2 days, then transferred into the same medium containing 200 mM NaCl or 20% PEG6000. The roots were sampled at 0, 6, 12, and 24 hours post-treatment (hpt).

For the different phytohormone treatments, three-week-old seedlings were uprooted and cultured in Hoagland liquid medium for 2 days, and then transferred into the same medium containing 100 μM methyl jasmonate (MeJA), 100 μM ethephon (ETH), 2 mM salicylic acid (SA) and 100 μM abscisic acid (ABA). The roots were sampled at 0, 6, 12, and 24 hpt. All samples were rapidly frozen in liquid nitrogen and stored at -70°C.

### Plasmid construction and *N*. *benthamiana* stable transformation

To overexpress *GmCYP82A3*, the coding DNA sequence (CDS) of *GmCYP82A3* (GenBank: NM_001254043.1) was amplified using the primers CYP82A3-F and CYP82A3-R from Conrad cDNA (S1 Table). The 1584 bp gene fragment was first cloned into the Gateway entry vector pDONR221, then cloned into pEarlyGate202 through an LR recombination reaction between the entry clone and the destination vector (Invitrogen, USA) [41]. The constructed vector was validated by sequencing, subsequently transformed into *Agrobacterium tumefaciens* (strain EHA105) by electroporation for further transformation.

The transgenic *N*. *benthamiana* plants were generated by *A*. *tumefaciens* mediated transformation from leaf discs, as described by Horsch [42]. The T1 seeds collected from self-pollinated T0 plants were germinated on MS medium with 50 mg/L Glufosinate ammonium (Sigma, USA) to produce T1 transgenic plants. T2 seeds were collected and the plants were cultured for functional characterizations. The transgenic plants were confirmed by PCR screening of both genomic DNA and cDNA using gene specific primers.

### Pathogen inoculation assay

We used several approaches to evaluate the effect of *GmCYP82A3* on plant resistance to pathogens. The uniform leaves cut from 7-week-old WT (wild type plant), EV (a transgenic line expressing empty vector as a negative control), and two *GmCYP82A3* overexpressing lines (2–3 and 4–1) were placed in Petri dishes, as described above for the soybean leaves. The detached leaves were infected with both necrotrophic *B*. *cinerea* and semi-biotrophic *P*. *parasitica*. For *B*. *cinerea* inoculation, we placed a 5 mm circular potato dextrose agar (PDA) agar containing mycelia cut from the edge of fresh cultured *B*. *cinerea* on the leaves. The diameters of the disease lesions were measured and the infected leaves were photographed at 4 days post-inoculation (dpi). For *P*. *parasitica* inoculation, the detached leaves were inoculated with 20 μl (approximately 5×10^4^ zoospores ml^-1^) of zoospore suspension. The WT and EV leaves were used as controls. The diameters of disease lesions were measured at 24 and 48 hpi, and the lesion area was calculated. Statistical significance was determined according to the Dunnett t-test method.

Two-week-old plants (T2 transgenic and WT) hydroponically grown in Hoagland liquid medium were inoculated with *P*. *parasitica* zoospore suspension. The roots were immersed in an approximately 5000 ml^-1^ zoospore suspension. The infected plants were then stored in the growth chamber and photographed at 2 and 5 dpi.

Seven-week-old plants were also used to determine the resistance level. One ml zoospore suspension (approximately 5×10^4^ zoospores ml^-1^) was dripped in a 1 ml deep hole close to the plant root for inoculation. Twenty plants from each line (WT, EV, 2–3 and 4–1) were infected. Plants with susceptible phenotypes were photographed at 2 and 5 dpi.

### Staining with trypan blue

To monitor cell death of *N*. *benthamiana* leaves and hypha growth of *P*. *parasitica*, the inoculated leaves were stained with lactophenol-trypan blue (10 ml of lactic acid, 10 ml of glycerol, 10 g of phenol, and 10 mg of trypan blue, dissolved in 10 ml of distilled water) [43]. After boiling for 5 min in the staining solution and de-staining in 2.5 g/ml chloral hydrate, the samples were mounted in 70% glycerol for microscopic observation.

### RNA isolation, semi-quantitative and quantitative real-time PCR

Total RNA was extracted using the Total RNA kit (Tiangen, CHINA), gDNA elimination and reverse transcription were performed with the PrimeScript^™^ RT reagent kit (TaKaRa, JAPAN). Quantitative real-time PCR was performed with the ABI PRISM 7500 real-time PCR system (Applied Biosystems, USA) using the AceQ^®^ qPCR SYBR Green Master Mix (Vazyme, CHINA) according to the manufacturer's instructions. The relative expression levels of target genes were calculated using the 2^–ΔΔCT^ method [44]. The significant difference of the genes expression was determined according to the Dunnett t-test method compared to WT. The genes encoded soybean *GmTubulin* alpha-3 (*GmTUA*) (GenBank: XM_006584532.1) [45] and *N*. *benthamiana NbEF1α* (GenBank: AY206004.1) [46] were selected as the references for soybean and *N*. *benthamiana*, respectively. A 476-bp specific sequence of *GmCYP82A3* was used to test transcription induction by *P*. *sojae* in soybean and transgenic *N*. *benthamiana* plants screened by RT-PCR. All the nucleic acid sequence of the primers list in S1 Table.

### Tolerance of transgenic *N*. *benthamiana* plants to abiotic stresses and JA

The seeds of WT, EV and two overexpression lines (2–3 and 4–1) were surface sterilized in 70% ethanol for 30 s, followed by 30% NaClO for 5 min, then washed at least five times with sterile distilled water. The seeds were placed in solid Murashige and Skoog (MS) medium with or without 100 mM NaCl or 8% PEG6000.

Germination rates were calculated from the percentage of seeds with radicles protruding through the seed coat. The assays were replicated at least three times, using 40 seeds each time. Seeds were geminated in the growth chamber with an 8D:16L photoperiod, 25°C, and 60% relative humidity.

For JA tolerance assays, the WT and transgenic tobacco plants (EV, 2–3, and 4–1) were germinated on MS medium containing 10 μM JA (Sigma, USA). The plates were placed in the chamber for 2 weeks to measure root elongation.

## Results

### *GmCYP82A3* highly expressed in resistant soybean cultivars and responds to *P*. *sojae* infection

**Fig 1:**
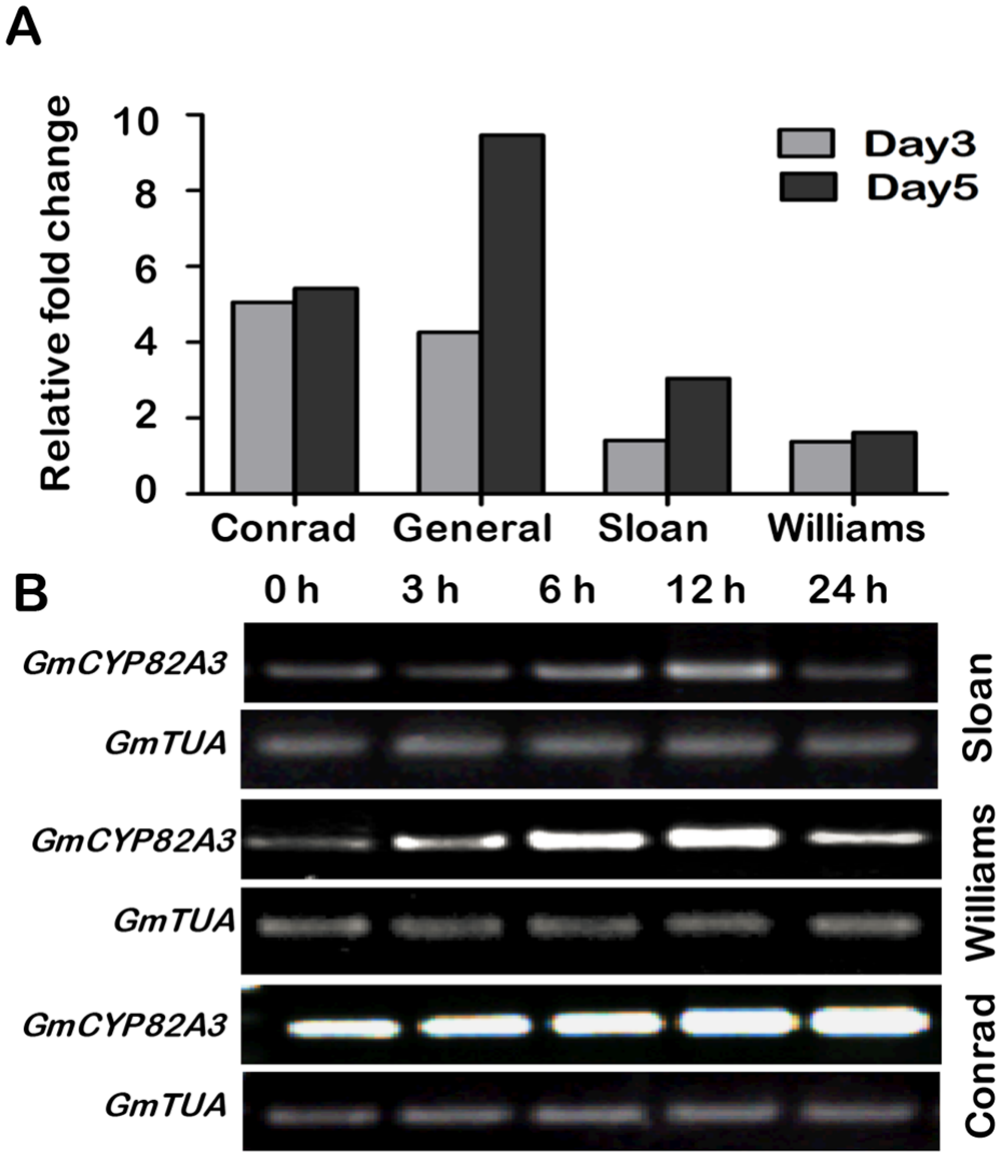
The expression pattern of *GmCYP82A3* during *P*. *sojae* infection. **(A)** The microarray data of relative expression levels of *GmCYP82A3*. The expression levels in four soybean cultivars with different resistance level (Conrad and General with highly partial resistance, Williams with moderately partial resistance and Sloan is highly susceptible). The relative expression levels were normalized to the mock samples. **(B)** Expression pattern of *GmCYP82A3* induced by *P*. *sojae* determined by RT-PCR. Soybean leaves of Sloan, Williams and Conrad were inoculated with *P*. *sojae* and samples were taken at 0, 3, 6, 12 and 24 hpi. The *GmTUA* gene serves as a control.

Gma.3136.2.A1_s_at was highly induced by *P*. *sojae* in cultivars with high level of partial resistance based on an Affymetrix GeneChip microarray data analysis [23]. This gene was up-regulated about 5-fold at both 3 and 5 dpi in Conrad, a cultivar with high level of partial resistance, and about 10-fold at 5 dpi in General, another highly partial resistant cultivar. However, the gene was weakly expressed in the moderately resistant cultivar Williams and the susceptible cultivar Sloan (Fig 1A).

Sequence analysis of this soybean EST showed that this gene is *GmCYP82A3* (GenBank: 359806337) and encodes a putative protein with 527-amino acids. GmCYP82A3 is a eukaryotic P450 protein, and shares 52% identity with cotton GhCYP82D1 [37], 49% and 48% identity with *Arabidopsis* AtCYP82C4 and AtCYP82C2 [29, 38] (S1 Fig).

To validate the response of *GmCYP82A3* to *P*. *sojae* infection, RT-PCR analysis was used to determine its expression profile in soybean leaves at 0, 3, 6, 12, and 24 hpi. *GmCYP82A3* was induced in Sloan, Williams and Conrad. In Sloan, *GmCYP82A3* was up-regulated after *P*. *sojae* infection, and reached the highest level at 12 hpi. In Williams, *GmCYP82A3* showed a similar expression pattern as in Sloan, but the expression level was much stronger. The transcription level was obviously up-regulated at 3 hpi, reached the highest level at 12 hpi, and was subsequently reduced at 24 hpi. The initial level in Conrad was much higher than in Williams, and continuously increased at later time points (Fig 1B).

### Expression of *GmCYP82A3* is induced by abiotic stresses and phytohormones

**Fig 2:**
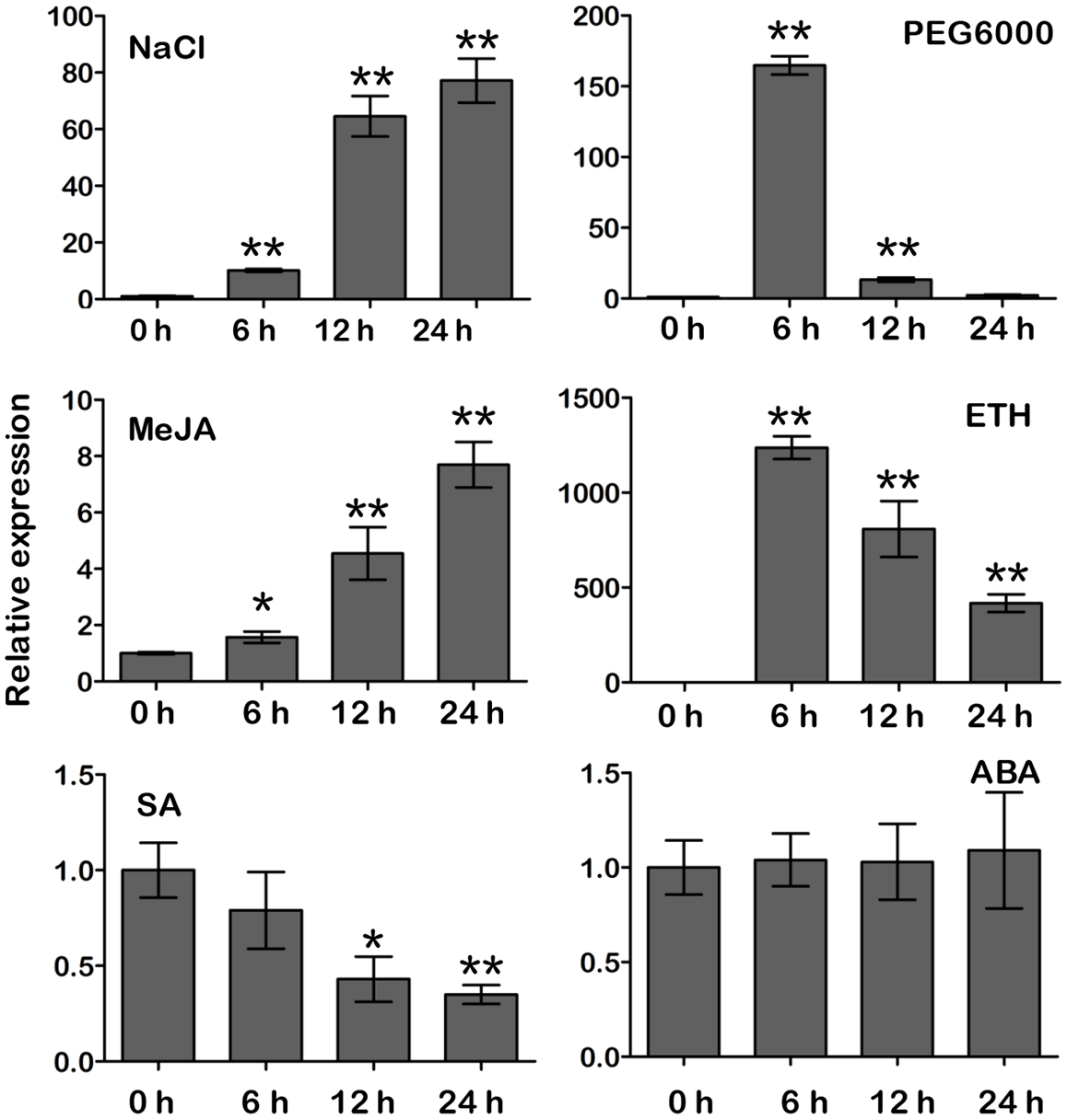
The expression profiles of *GmCYP82A3* response to various abiotic stress and phytohormones. Three-week-old seedlings were treated with the indicated compounds, and then the samples were collected at 0, 6, 12 and 24 hpt. The relative expression level was normalized to soybean *GmTUA*. Mean and standard deviation (SD) were calculated from three independent biological replicates. The asterisk at the top of the columns indicate significant differences (Dunnett-t test, * *P*<0.05, ** *P*<0.01).

Then, we characterized the expression patterns of *GmCYP82A3* response to abiotic stresses in Conrad, using qRT-PCR. In general, the expression of *GmCYP82A3* was dramatically up-regulated in both PEG6000 and NaCl treatments, but with different trends. When treated with NaCl, the expression level was up-regulated 10-fold at 6 hpt and 64-fold at 12 hpt, then maintained high levels at subsequent time points. When treated with PEG6000, expression was up-regulated 164-fold at 6 hpt and then dramatically reduced at later time points (Fig 2).

Previous studies showed that some *CYP82* family genes that share high identity with *GmCYP82A3* could be induced by phytohormones [37, 38]. To examine responses to phytohormones, soybean seedlings of Conrad were treated with methyl jasmonate (MeJA), ethephon (ETH), salicylic acid (SA), and abscisic acid (ABA). As shown in Fig 2, expression levels were enhanced at all time points after MeJA treatment, and up-regulated about 8-fold at 24 hpt. However, it was dramatically up-regulated by more than 1,000-fold at 6 hpt, and then attenuated, but still maintained a high level with ETH treatment. In contrast, a decrease in *GmCYP82A3* expression was observed when treated with SA, and there was no response to ABA treatment.

### *GmCYP82A3* overexpression enhances resistance of *N*. *benthamiana* to *B*. *cinerea* and *P*. *parasitica*

**Fig 3:**
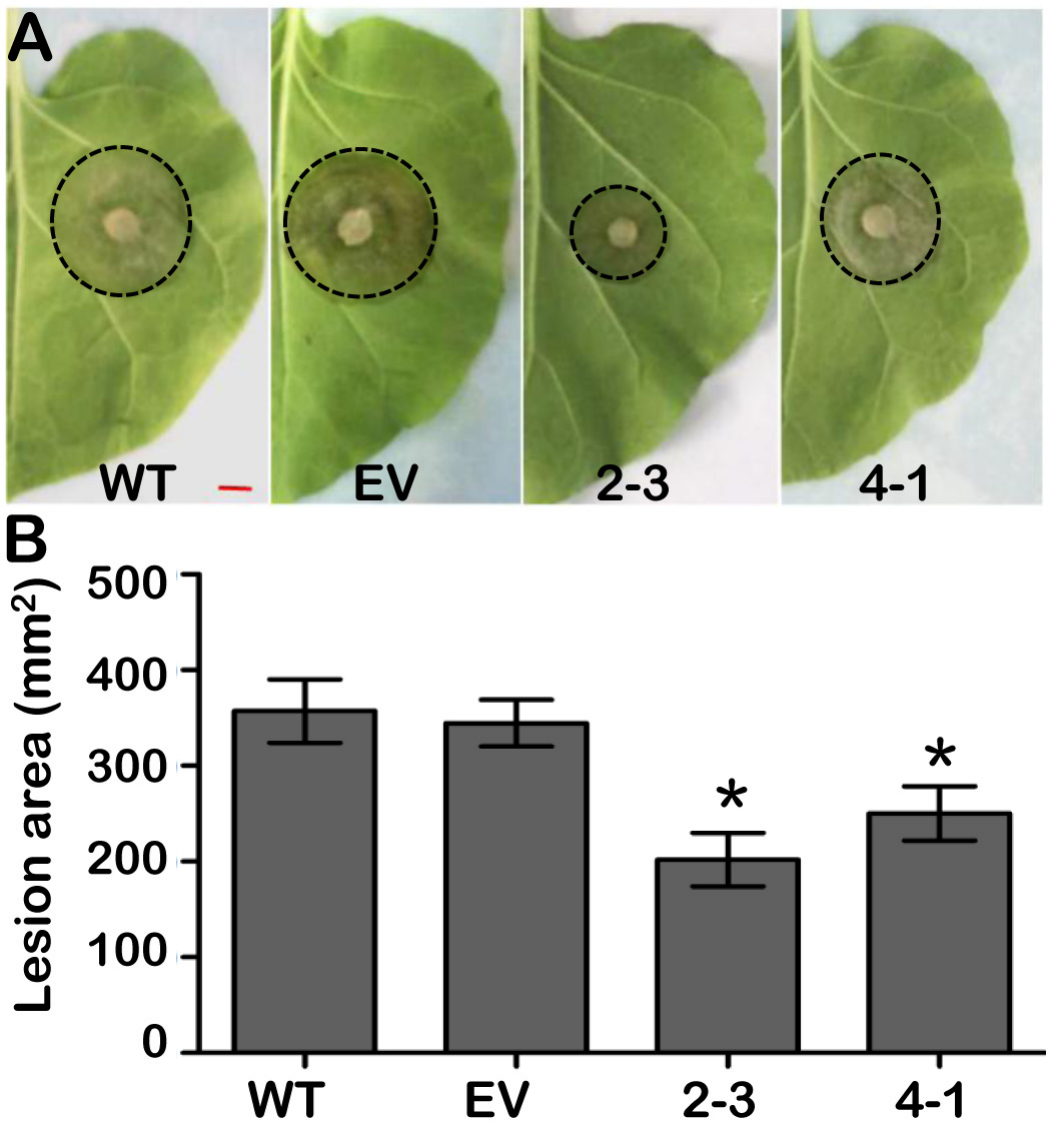
Enhanced resistance to *B*. *cinerea* of transgenic *N*. *benthamiana* lines. **(A)** Phenotypes of the *N*. *benthamiana* leaves from WT, EV and overexpression lines (2–3 and 4–1) inoculated with *B*. *cinerea*. The mycelia growing on PDA medium was used to infect the transgenic and wild type leaves. Photographs were taken 4 dpi. Bar = 5 mm. **(B)** Lesion area of inoculated leaves. Lesion diameters were measured at 4 dpi and then the lesion area was calculated. Similar results were observed at least three duplications. SD represented with the bars (Dunnett-t test: * *P*<0.05).

To explore the role of this *P450* family gene, *GmCYP82A3* was overexpressed in *N*. *benthamiana* plants driven by the 35S promoter and two independent T2 generations were selected for functional characterizations. Transgene integration and expression were confirmed by genomic PCR and RT-PCR, respectively (S2 Fig). No obvious phenotypic differences were observed between the WT, EV, and *GmCYP82A3* overexpressing (2–3 and 4–1) seedlings. We first challenged the detached leaves with *B*. *cinerea*, a necrotrophic pathogen and causes necrotic symptoms on *N*. *benthamiana* (Fig 3A, S3 Fig). The average sizes of the lesion areas were ~ 357.15 and ~ 344.54 mm^2^ at 4 dpi in WT and EV leaves, respectively. However, the necrotic areas were significantly reduced in the two independent overexpression lines. The average sizes of the lesion areas were ~ 201.97 and ~ 250.12 mm^2^ (Fig 3B). The results showed that *GmCYP82A3* could enhance resistance to *B*. *cinerea*.

**Fig 4:**
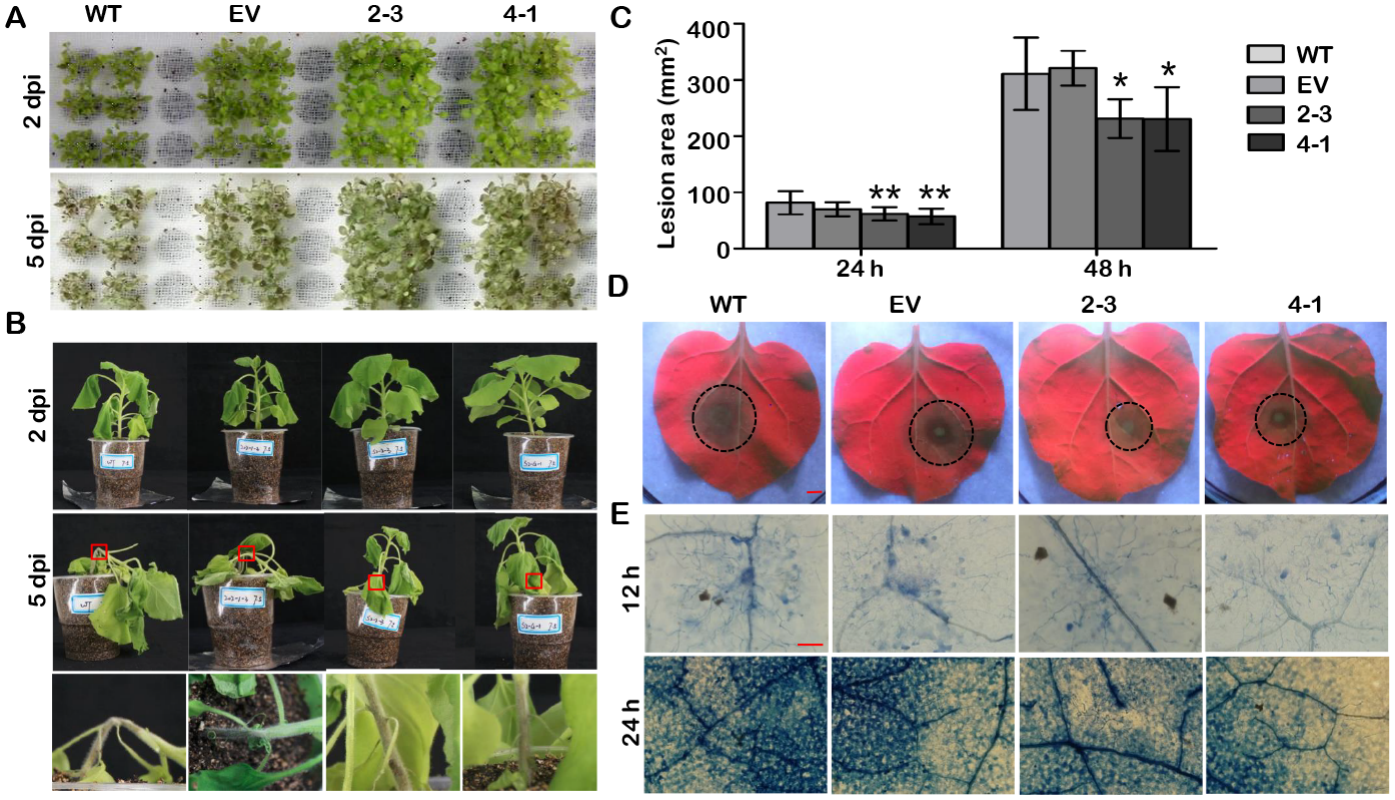
Increased resistance to *P*. *parasitica* of transgenic *N*. *benthamiana* lines. **(A)** Phenotypes of the 2-week-old *GmCYP82A3* transgenic plants inoculated with *P*. *parasitica* zoospores. Photographs were taken at 2 and 5 dpi, respectively. **(B)** Phenotypes of the 7-week-old *GmCYP82A3* transgenic plants inoculated with *P*. *parasitica* zoospores. Photographs were taken at 2 and 5 dpi, respectively. The photos of the bottom row are high magnification views of the red pane marked in the upper row. **(C)** Lesion area of the inoculated leaves measured at 24 and 48 hpi. The lesion area was calculated from over three repeats. SD represented with the bars (Dunnett-t test: * *P*<0.05, ** *P*<0.01). **(D)** Detached leaves from WT, EV and *GmCYP82A3* overexpression plants were inoculated with *P*. *parasitica* zoospores. Photographs were taken at 48 hpi under a UV lamp. Bar = 5 mm. **(E)** Trypan blue staining of the *P*. *parasitica* inoculated sites of *N*. *benthamiana* leaves. The hypha accumulation in the WT, EV and *GmCYP82A3* overexpression leaves were detected using trypan blue staining at 12 and 24 hpi. Bar = 100 μm.

To further study the disease resistance function of *GmCYP82A3*, we also characterized the resistance level of transgenic plants to *P*. *parasitica*, a hemibiotrophic pathogen that is similar to *P*. *sojae*. When the hydroponically cultured 2-week-old plants were inoculated with *P*. *parasitica* zoospores, the WT and EV plants withered at 2 dpi, then exhibited stem rot and collapse of the whole plant at 5 dpi. In contrast, the two overexpression lines were more resistant and the symptoms were much weaker (Fig 4A).

The root inoculation assay performed on 7-week-old plants to confirm the resistant phenotype. Inoculated seedlings displayed wilting symptoms at 2 dpi. However, the brown and necrotic areas on stems progressively expanded from the roots, causing stem wither and lodge in WT and EV plants at 5 dpi. Symptoms were restricted or expanded slightly in the two overexpression plant lines during the same period (Fig 4B).

The detached transgenic leaves were also used to test for resistance. The average lesion areas caused by *P*. *parasitica* zoospores infection were significantly smaller on *GmCYP82A3* overexpression leaves than those on WT and EV leaves, at both 24 and 48 hpi (Fig 4C and 4D). Trypan blue staining showed fewer hyphae on *GmCYP82A3* overexpression leaves (Fig 4E). These results imply that *GmCYP82A3* contributes to resistance to the two tested pathogens.

### *GmCYP82A3* overexpression *N*. *benthamiana* plants are insensitive to JA

**Fig 5:**
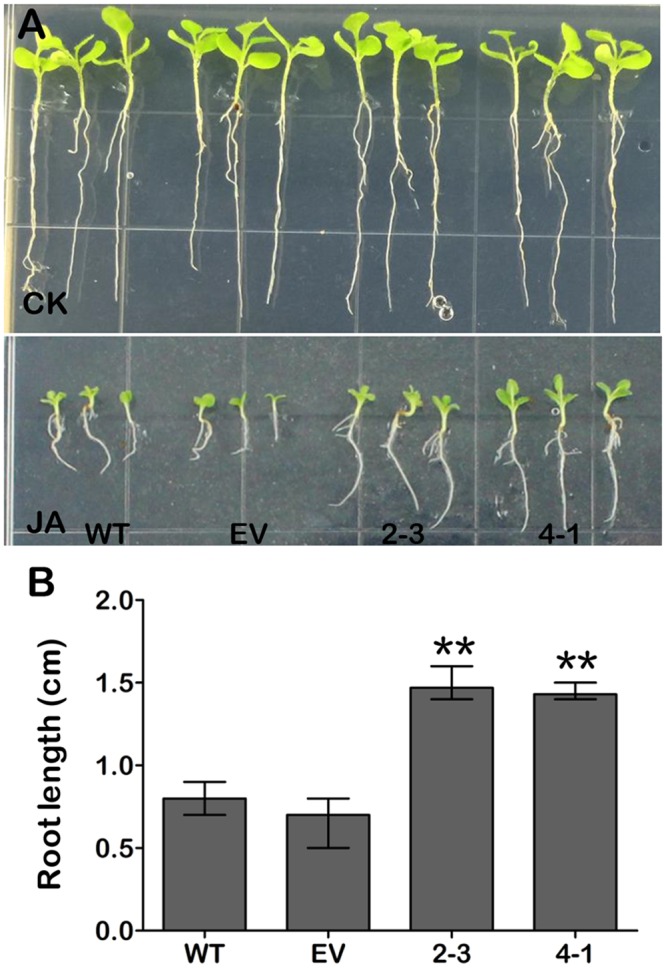
The effect of *GmCYP82A3* on root growth under JA treatment. **(A)** The phenotypes of 2-week-old seedlings of indicated genotypes grown on MS medium without (Control) or with 10 μΜ JA. **(B)** Root length of 2-week-old WT, EV and *GmCYP82A3* overexpression seedlings grown on MS medium containing 10 μΜ JA. The experiments were repeated three times with similar results. SD represented with the bars. The asterisk at the top of the columns indicate significant differences (Dunnett-t test, ** *P*<0.01).

JA plays an important role in plant defense signaling pathway and it also inhibits root growth [47]. Both *GhCYP82D1*-silenced cotton and *AtCYP82C2* mutant *Arabidopsis* seedlings were hypersensitive to exogenous JA treatment [37, 38]. We evaluated whether *GmCYP82A3* could alter the sensitivity of plants to JA. Under normal growth conditions, the overall growth rate and morphology of *GmCYP82A3* overexpression plants were similar to WT and EV plants. However, when 10 μM JA were added to the medium, roots of the two selected lines were significantly longer than those of WT and EV roots (Fig 5), suggesting that the plants expressing *GmCYP82A3* were less sensitive to JA-mediated inhibition of root growth. The results above imply that *GmCYP82A3* might be involved in the JA signaling pathway.

### *GmCYP82A3* affects the expression of defense related marker genes

**Fig 6:**
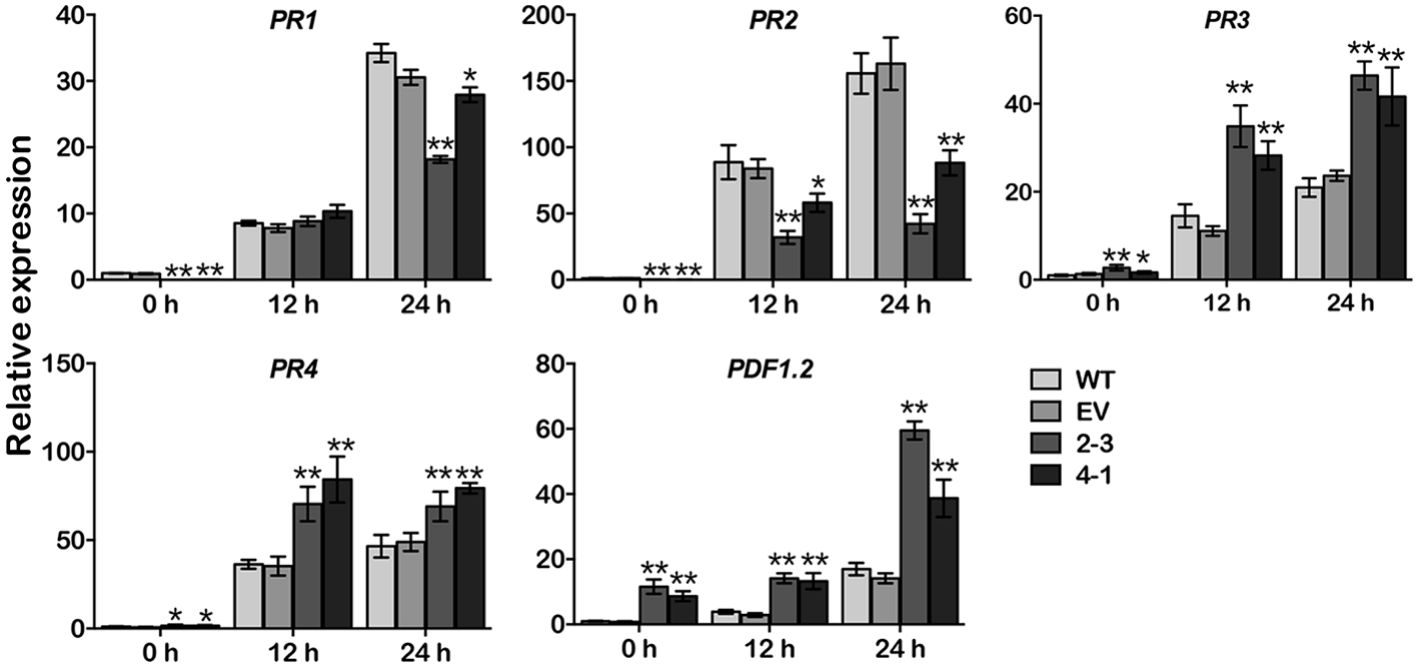
Expression profiles of defense marker genes in transgenic *N*. *benthamiana* plants. Total RNA was extracted from detached leaves of WT, EV and *GmCYP82A3* overexpression plants at 0, 12, 24 hpi by *P*. *parasitica* zoospores. Expression levels were determined by qRT-PCR using gene specific primers and normalized to *NbEF1a* with three replicate experiments. Data are the means of three replications, error bars indicate SD. The significant differences between WT and transgenic plants are indicated by asterisk (Dunnett-t test, * *P*<0.05, ** *P*<0.01).

Because of changes of resistance levels to *P*. *parasitica* infection, we investigated *GmCYP82A3* overexpression plants for the expression pattern of defense related marker genes in the defense cascade, and to gain further insight into possible regulation pathways. Four well known *PR* (*pathogenesis related*) genes were firstly selected for comparison. The SA marker *PR1* (*Pathogenesis-related protein 1*) and *PR2* (*Pathogenesis-related protein 2*) [48] were significantly lower in *GmCYP82A3* overexpression plants than those in WT and EV plant before and after infection, except *PR1* showed no different at 12 hpi. In contrast, the basal expression levels of another two genes response to JA/ET, *PR3* (*B-chitinase*) and *PR4* (*Hevein-like protein*) [49], were significantly higher in the two overexpression lines than in EV and WT plants. Both *PR3* and *PR4* greatly increased after infection with *P*. *parasitica*, and also had a higher expression level in overexpression plants. JA and ET regulated defense related marker gene *PDF1*.*2* (*Plant defensing 1*.*2*) [49] significantly increased in overexpression plants and the basal expression levels were about 11 and 8-fold higher in 2–3 and 4–1 lines than that in WT. Meanwhile, *PDF1*.*2* expression induced by *P*. *parasitica* infection was also significantly higher in overexpression plants at 12 and 24 hpi (Fig 6).

### *GmCYP82A3* is involved in the JA/ET signaling pathway

For further detecting the signaling transduction effected by *GmCYP82A3*, several key regulators in the JA/ET signaling pathways were selected for expressional analysis. *LOX1* (*Lipoxygenase 1*) is involved in JA biosynthesis and signaling pathway [50]. Its basal expression was not effected between WT and transgenic plants, but the expression exhibited more intense inductive effect after infection in overexpression plants. JAR1 (Jasmonate resistant 1) catalyzes JA conjugated to isoleucine, resulting in biologically highly active specific enantiomer of jasmonoyl-isoleucine (JA-Ile) [51]. *COI1* (*Coronatine insensitive 1*) encodes an F-box protein to assemble SCF^COI1^ protein complex, act as JA-Ile receptor in JA signaling [52]. In the expressional analysis, *JAR1* was induced at 12 hpi and then reduced at 24 hpi, although, the expression levels were significantly higher in overexpression plants both before and after infection. The expression of *COI1* down-regulated after infection, and there was no difference between the lines at both 0 and 12 hpi, but up-regulated and significantly higher in overexpression lines at 24 hpi.

**Fig 7:**
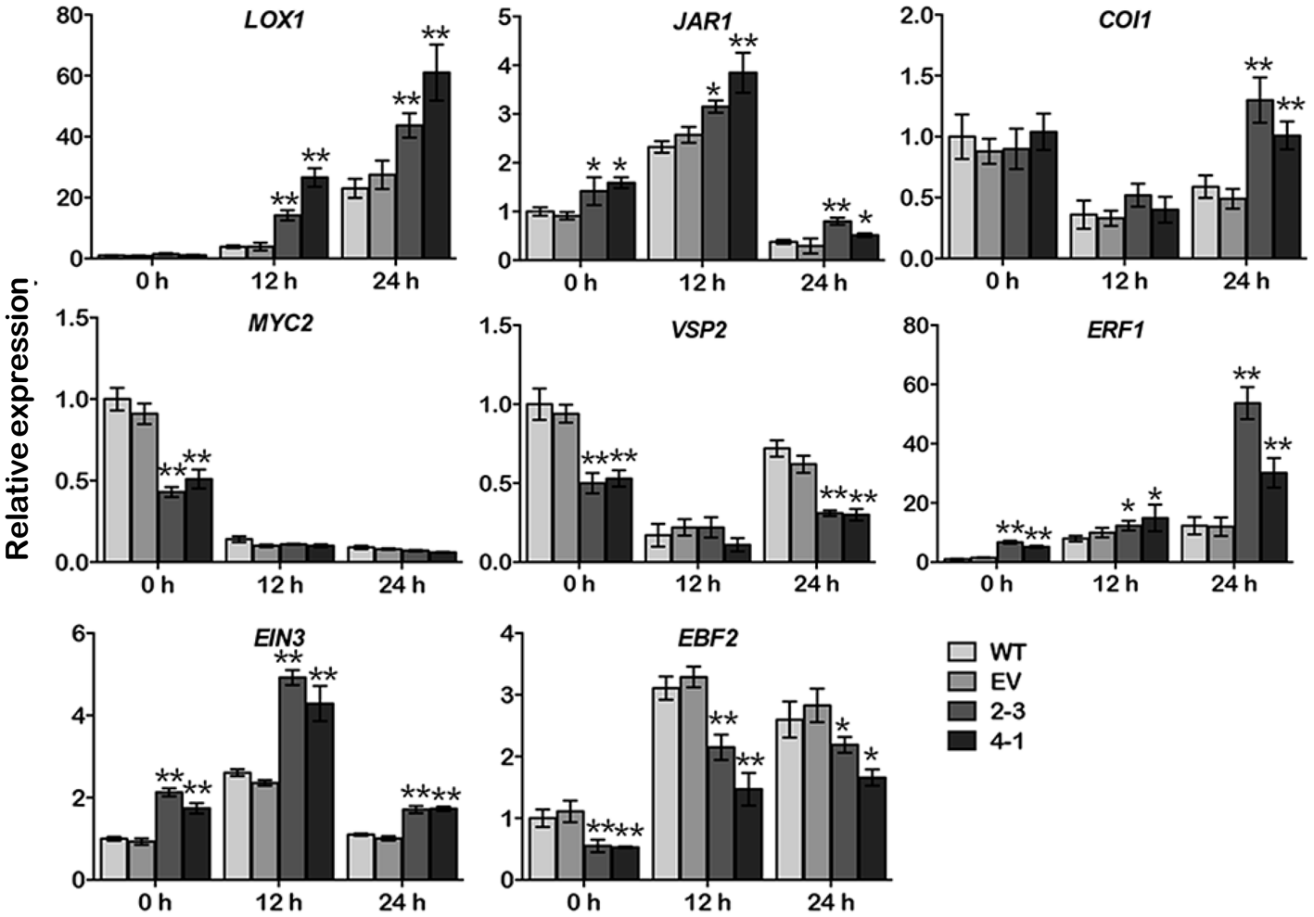
Expression profiles of key JA/ET signaling pathway genes in transgenic *N*. *benthamiana* plants. Data are the means of three replications, error bars indicate SD (Dunnett-t test, * *P*<0.05, ** *P*<0.01).

The downstream of JA signaling occurs via two different branches, which regulated by *MYC2* (*Myelocytomatosis protein 2*) or *ERF1* (*Ethylene response factor 1*) respectively [49]. In this study, *MYC2* and *VSP2* (*Vegetative storage protein 2*) [53] selected as the MYC branch marker genes. The results indicated that the basal expression of *MYC2* and *VSP2* were suppressed in *GmCYP82A3* overexpression plants. After infection, both genes were decreased, and *MYC2* was no difference between overexpression and control plants, but VSP2 was significantly lower in overexpression plants at 24 hpi. In ERF branch, *ERF1* plays a crucial role in the cross talk between JA and ET signaling pathway [54, 55], the expression was induced by *P*. *parasitica* infection. In overexpression plants, the expression of *ERF1* showed significantly higher both before and after infection (Fig 7).

In ET signaling pathway, *EIN3* (*Ethylene insensitive 3*) positive regulate downstream transcription of ethylene response [56], the expression was induced at 12 hpi and then reduced at 24 hpi, and exhibited significantly higher in overexpression plants than that in EV and WT plants. EBF2 (Ein3-binding F box protein 2) which regulate EIN3 protein degradation [56], the expression was suppressed both before and after infection compared with WT and EV. (Fig 7). These results indicated that overexpression *GmCYP82A3* disturbed the key regulate genes expression, suggesting that *GmCYP82A3* might be involved in the JA/ET-mediated signaling pathway and contributes to delayed disease development when challenged with *P*. *parasitica* and *B*. *cinerea* on detached leaves.

### *GmCYP82A3* overexpression enhances seed germination under salt and osmotic stress

**Fig 8:**
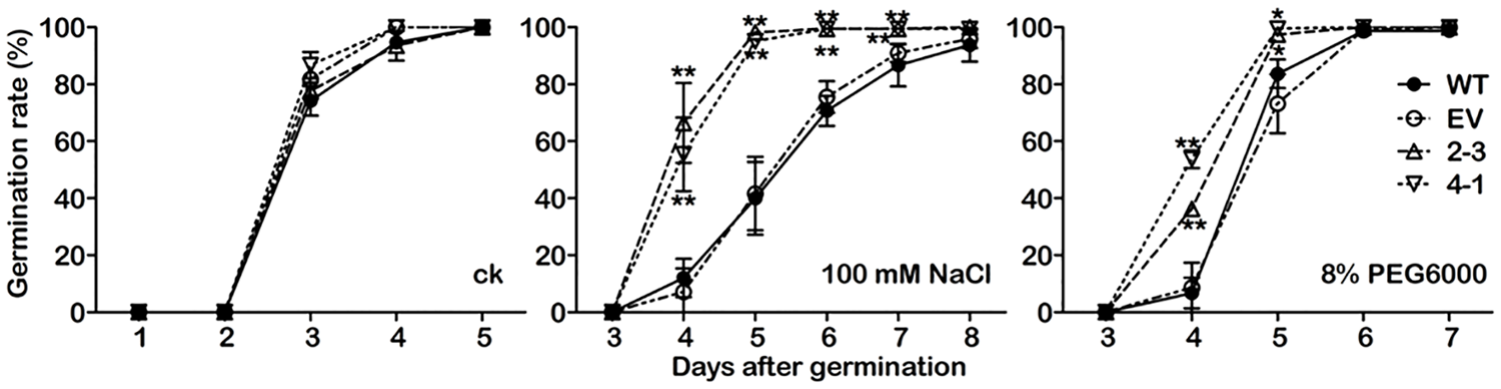
Seed germination rates of transgenic *N*. *benthamiana* under abiotic stress. The seeds as indicated were cultured on MS medium (**A**) or containing NaCl (**B**) or PEG6000 (**C**). The germination rates were calculated from the percentage of seeds with radicle protruded through the seed coat after cultivation every day. Mean values and SD were obtained from five independent experiments (n = 40). The significant differences between WT and transgenic plants are indicated by asterisk (Dunnett-t test, * *P*<0.05, ** *P*<0.01).

Given that the expression of *GmCYP82A3* was significantly induced by salt and drought stress (Fig 2), we conducted tests to determine whether *GmCYP82A3* could regulate the response of the transgenic plants to abiotic stresses. Germination rates of the transgenic and WT seeds were calculated under abiotic stress. The germination of WT, EV, and overexpression seeds showed no obvious differences on the MS medium without NaCl or PEG6000 (Fig 8A). Under treatment with 100 mM NaCl and 8% PEG 6000, germination rates of the seeds showed significant difference. The germination of WT and EV seeds were severely suppressed under the stresses. However, the germination rates of *GmCYP82A3* overexpression seeds were higher compared with the WT and EV seeds at 4–7 and 4–5 days after sowing on MS medium containing 100 mM NaCl and 8% PEG 6000 respectively (Fig 8B and 8C). Imply that enhanced tolerance to salt and drought stress of *GmCYP82A3* overexpression seeds.

## Discussion

Since partial resistance is important for soybean breeding resistance to Phytophthora root and stem rot, attempts have been made to identify the functional candidate genes conferring this resistance. A wide list of candidate genes underlying the soybean QTL conferring resistance to *P*. *sojae* were identified by whole-genome transcription profiling analysis of cultivars with different levels of resistance [13, 14, 20]. In this study, we performed a functional analysis of the soybean *P450* gene, called *GmCYP82A3*, which is highly expressed in partial resistant soybean cultivars during *P*. *sojae* infection and underlying the QTL conferring resistance to *P*. *sojae* [14]. Our results indicated that the expression level of *GmCYP82A3* can be induced by various abiotic stresses and phytohormones molecules. The overexpressed transgenic *N*. *benthamiana* plants showed enhanced resistance to the necrotrophic pathogen *B*. *cinerea* and the hemibiotrophic pathogen *P*. *parasitica*, implying that this gene underlying the QTL region may contribute to plant resistance. Further examination found the transgenic plants were less sensitive to jasmonic acid (JA), and the enhanced resistance accompanied with increased expression of the JA/ET signaling pathway genes.

Previous studies revealed that members of CYP82 family are highly stress responsive in tobacco, pea, soybean, and *Arabidopsis* [28–31]. In this study, *GmCYP82A3* was induced by *P*. *sojae* infection and showed a different transcription profiles in soybean cultivars with diverse partial resistant levels, which consists with that from the candidate genes identification of soybean partial resistant QTL [13]. Besides the pathogen induction, *GmCYP82A3* was remarkably up-regulated by salt and drought treatment. Phytohormones are widely believed to play key roles in signaling transduction involved in plant responses biotic and abiotic stresses [57–59]. *GmCYP82A3* was highly induced by MeJA and ETH, but reduced in some degree when treated with SA, and did not respond to ABA treatment. This was consistent with previous reports, in which cotton and *Arabidopsis CYP82* genes were both highly induced by JA/MeJA treatment [37, 38]. Thus, we speculated that this soybean *P450* gene may play important role in abiotic stress and defense processes.

JA, ET, SA signaling and the cross-talk between them play a role in plant defense response activation upon pathogen infection. For example, SA mediates defense responses to biotrophic pathogens, JA/ET mediates defense against necrotrophic or hemibiotrophic pathogens and usually antagonism with SA [58, 59]. Transcriptional analysis of phytohormone signaling marker genes revealed that *GmCYP82A3* overexpression affected the phytohormone signaling transduction. Two SA signaling marker genes, *PR1* and *PR2* were suppressed in transgenic plants both before and after pathogen infection compare with control, especially *PR2*. The results proved that *GmCYP82A3* partly suppressed SA signaling pathway. *PR3*, *PR4* and *PDF1*.*2*, which involved in JA and ET dependent resistance were significantly higher expression levels in overexpression plants [49]. We speculated that *GmCYP82A3* might be involved in the JA/ET signaling pathway. In this study, the root growth in response to JA of *GmCYP82A3* overexpression plants showed a similar phenotype of *AtCYP82C2*, which the *Arabidopsis* overexpression plants also showed less sensitive to JA-mediated root growth inhibition [38], implying that *GmCYP82A3* is involved in JA signaling. *LOX1* has been widely demonstrated to be involved in JA biosynthesis and signaling pathway [50], JAR1 catalyzes the formation of a biologically active JA-Ile conjugate [51]. *COI1* encodes an F-box protein to assemble SCF^COI1^ protein complex, act as JA-Ile receptor in JA signaling. The binding of JA-Ile to COI1 leads degradation of JASMONATE ZIM-domain (JAZ) transcriptional repressor proteins, and results in the activation of JA responsive genes [52]. The higher expression levels of *LOX1*, *JAR1*, and *COI1* in *GmCYP82A3* overexpression plants after *P*. *parasitica* infection suggest that the JA signaling transduction may be enhanced. At least two distinct branches lie in JA signaling pathway to regulates downstream genes expression [49, 53]. ERF branch confer resistance to necrotrophic pathogens and MYC2 branch is associated with wound response and insect herbivores resistance, but MYC2 branch also has been demonstrated to play a role for enhance pathogen defense [49, 60, 61]. Alternatively, the MYC branch has an antagonistic effect with ERF branch, *AtMYC2* represses the expression of ERF branch genes while activating the wound responsive genes such as *VSP2* in MYC branch [53]. The ERF branch and downstream defense relate genes *ERF1*, *PDF1*.*2*, *PR3* and *PR4* showed significant up-regulated in *GmCYP82A3* overexpression plants. In contrast, the expression of *MYC2* and *VSP2* which involved in the MYC branch were suppressed. The results demonstrated that ectopic expression *GmCYP82A3* enhanced the signaling transduction of JA ERF branch during the transgenic *N*. *benthamiana* plants and *P*. *parasitica* interaction.

Ethylene regulates wide physiological responses in plants. Ethylene is the first plant hormone, the signaling pathway has provided a framework in *Arabidopsis*. EIN3 is a plant specific nuclear transcription factor and positive regulate downstream transcription of ethylene response, such as the target gene of *ERF1* [55, 56]. The critical regulatory mechanism of ethylene signaling in the nucleus is controlled by EIN3 protein levels. In the absence of ethylene, EIN3 is rapidly degraded by 26S proteasomal under the regulation of two F-box proteins EBF1 and EBF2. In the presence of ethylene, EBF1 and EBF2 are degraded, thus allowing EIN3 protein accumulation and activated ethylene responsive gene expression [56]. In this study, transcription profiles of *EIN3* during the pathogen infection was elevated in *GmCYP82A3* overexpression plants, whereas, *EBF2* showed suppression effect. In *Arabidopsis*, the ET and ERF branch of JA signaling act synergistically on the defense related genes expression, such as *PDF1*.*2*, *ERF1*, *PR3* and *PR4* [54, 62–64]. *ERF1* transcript was induced by *B*. *cinerea* infection, and the overexpressed *Arabidopsis* plants showed enhanced resistance to necrotrophic fungi such as *B*. *cinerea* and *Plectospaerella cucumerina*, but reduced tolerance to biotrophic *Pst* DC3000 [65]. We believed that the ET signaling transduction was activated and contributed to the enhanced resistance.

Besides play essential roles in regulating plant defense against pathogens as discussed above, JA/ET signaling also contributed to plant abiotic stress tolerance such as salt and drought [57]. Transgenic expression of several genes involved in JA biosynthesis and signaling pathway showed enhanced salt tolerance [66–68]. The pathway component EIN3 performed as a positive regulator on salt stress tolerance, further studies found the downstream ERF1 selectively actives salt tolerance genes by binding to the DRE-box of these genes promoter [69, 70]. Drought tolerance can be mediated through a wide range of mechanisms. Till now, JA and ET signaling pathways are also implicated in drought tolerance [57].

In this study, ectopic expression soybean *GmCYP82A3* in *N*. *benthamiana* enhanced resistance to two different kinds of pathogens and salt, drought stress tolerance. The transcription analysis of phytohormones pathway genes revealed that *PR1* and *PR2* involved in SA signaling was suppressed, on the other hand, the ERF branch of JA and ET signaling genes were upregulated in the transgenic plants, indicating that the ERF branch of JA and ET signaling pathways were activated by *GmCYP82A3*. But the potential mechanism is still needed to be explored. Since several CYP82 family members participate in a variety of metabolic pathways [32–38], so further characterization of the biochemical function is needed.

As a form of incomplete resistance in the *P*. *sojae*-soybean system and non-race-specific resistance, partial resistance has been proposed as a way to improve the breeding efforts for soybean resistance to *P*. *sojae*. Previous studies showed that some cultivars with high levels of partial resistance were difficult to distinguish from the *Rps* genotype when inoculated with the simple isolate [71]. In this study, we found that *GmCYP82A3* affected defense related genes expression associated with the JA/ET signaling pathway, conferring transgenic plants resistant to two different types of pathogens. From our understanding of the mechanism of soybean partial resistance, we believe that the strategy of generate highly partial resistant soybean cultivars offer a promising approach for Phytophthora root rot control.

## Supporting Information

S1 Table Sequences of the gene-specific primer pairs used in this study. (DOCX)

S1 Fig Sequence alignment of GmCYP82A3 with GhCYP82D1, AtCYP82C4 and AtCYP82C2. Sequence alignment was done by ClustalW. Black and gray backgrounds indicate identical and similar residues, respectively; dotted lines indicate gaps.
(TIF)

S2 Fig Molecular detection of transgenic *N*. *benthamiana* lines. Electrophoresis pattern of PCR from genomic DNA (**A**) and cDNA (**B**) corresponding to *GmCYP82A3* (upper panel) of wild-type (WT), empty vector (EV) transformants (EV-1, EV-2) and the six T2 transgenic lines expressing *GmCYP82A3* (2–3, 3–3, 4–1, 10–1, 15–2, and 9–1). The *NbEF1a* (lower panel) was used as an internal control. M, DNA Marker DL2000 PLUS.
(TIF)

S3 Fig Preliminary study of the resistant levels of transgenic *N*. *benthamiana* lines to *B*. *cinerea*. **(A)** Phenotypes of the *N*. *benthamiana* leaves from WT, EV and overexpression lines (2–3 and 4–1) inoculated with *B*. *cinerea* at 4 dpi. Bar = 5 mm. **(B)** Lesion area of inoculated leaves. Lesion diameters were measured at 4 dpi and then the lesion area was calculated. SD represented with the bars (Dunnett-t test: ** *P*<0.01).
(TIF)
