# Supplementary material for: GmCYP82A3, a Soybean Cytochrome P450 Family Gene Involved in the Jasmonic Acid and Ethylene Signaling Pathway, Enhances Plant Resistance to Biotic and Abiotic Stresses
Source: PLoS One. 2016 Sep 2;11(9):e0162253. doi: 10.1371/journal.pone.0162253 (PMC5010195; doi:10.1371/journal.pone.0162253)
Supplement: S1 Table — (DOCX) [file pone.0162253.s001.docx]

**S1 Table Sequences of the gene-specific primer pairs used in the study**

| Primer name | Primer sequence |
| --- | --- |
| CYP82A3-F | 5'- GGGGACAAGTTTGTACAAAAAAGCAGGCTTCACCATGGACCTTCTCCTAAATTGCCTA-3' |
| CYP82A3-R | 5'-GGGGACCACTTTGTACAAGAAAGCTGGGTTTTATAAAGTTTCATAATAGTTGGG-3' |
| CYP82A3Test-F | 5'-ACCTTGCCGTTTCATCTCG-3' |
| CYP82A3Test-R | 5'-TGCCTTCATAGCCTTCTCA-3' |
| GmTUA-F | 5'-ACCATCAAGACTAAGAGGACTG-3' |
| GmTUA-R | 5'-AACAAAAAGGAACGAACAATAC-3' |
| NbEF1a-F | 5'-AGAGGCCCTCAGACAAAC-3' |
| NbEF1a-R | 5'-TAGGTCCAAAGGTCACAA-3' |
| PR1-F | 5'-GTGGACACTATACTCAGGTG-3' |
| PR1-R | 5'-TCCAACTTGGAATCAAAGGG-3' |
| PR2-F | 5'-AGGTGTTTGCTATGGAATGC-3' |
| PR2-R | 5'-TCTGTACCCACCATCTTGC-3' |
| PR3-F | 5'-CAATGCCTTTATCAATGCTG-3' |
| PR3-R | 5'-AGTAGTCACCTGGGCTACCT-3' |
| PR4-F | 5'-GATGCTTGAGGGTGACGA-3' |
| PR4-R | 5'-ATAGCCCACTCCATTTGT-3' |
| PDF1.2-F | 5'-CTTCAAGCAAAGCTGCAGCCAAAG-3' |
| PDF1.2-R | 5'-CTATGCACTAAGCCATGTGTGTTTG-3' |
| LOX1-F | 5'-AAAACCTATGCCTCAAGAAC-3' |
| LOX1-R | 5'-ACTGCTGCATAGGCTTTGG-3' |
| JAR1-F | 5'-GAGTGAACTTTAACCCAA-3' |
| JAR1-R | 5'-CAGTAAGACCCACAGGAG-3' |
| COI1-F | 5'-GAACAGGAAATGGAGGAC-3' |
| COI1-R | 5'-AGTAGAACCAACCGAAAA-3' |
| MYC2-F | 5'-GAAGCGGATAGTAGTAGAGTT-3' |
| MYC2-R | 5'-TTTCTCCCTCCTTTGTCT-3' |
| VSP2-F | 5'-CCCTCCTTTCCACTGTCC-3' |
| VSP2-R | 5'-GTCCCTCAATGCTACTCCAT-3' |
| ERF1-F | 5'-GCTCTTAACGTCGGATGGTC-3' |
| ERF1-R | 5'-AGCCAAACCCTAGCTCCATT-3' |
| EIN3-F | 5'-TCACTCGGAAGAGGAAGC-3' |
| EIN3-R | 5'-TGCGGACATTGAAGACAC-3' |
| EBF2-F | 5'-GGAGCAAGAGCCATAACA-3' |
| EBF2-R | 5'-TTCCCTTCCACAGACCTA-3' |
