# Supplementary figures and images for: GmCYP82A3, a Soybean Cytochrome P450 Family Gene Involved in the Jasmonic Acid and Ethylene Signaling Pathway, Enhances Plant Resistance to Biotic and Abiotic Stresses
Source: PLoS One. 2016 Sep 2;11(9):e0162253. doi: 10.1371/journal.pone.0162253 (PMC5010195; doi:10.1371/journal.pone.0162253)

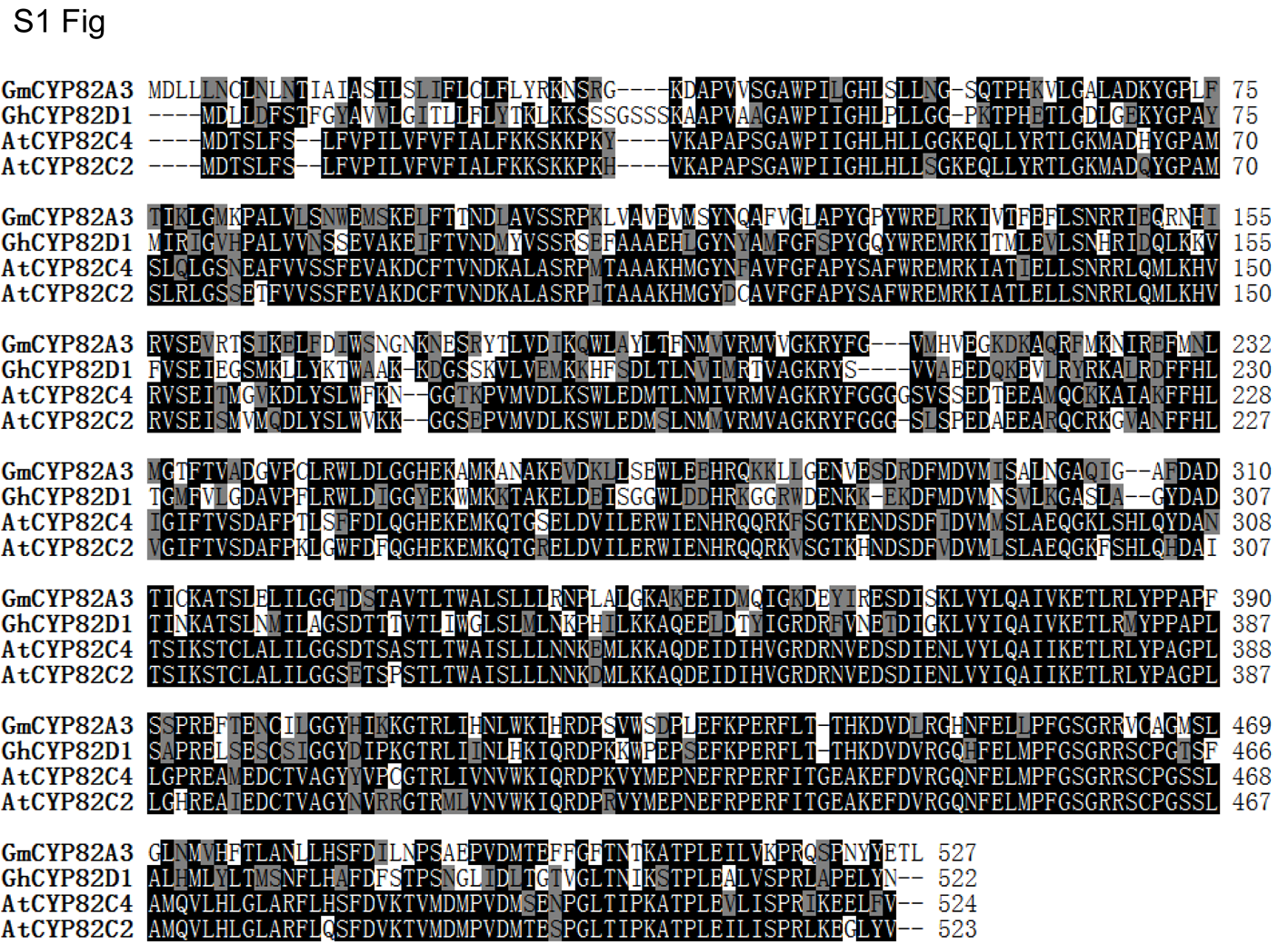

Supplement: S1 Fig — Sequence alignment was done by ClustalW. Black and gray backgrounds indicate identical and similar residues, respectively; dotted lines indicate gaps. (TIF) [file pone.0162253.s002.tif]

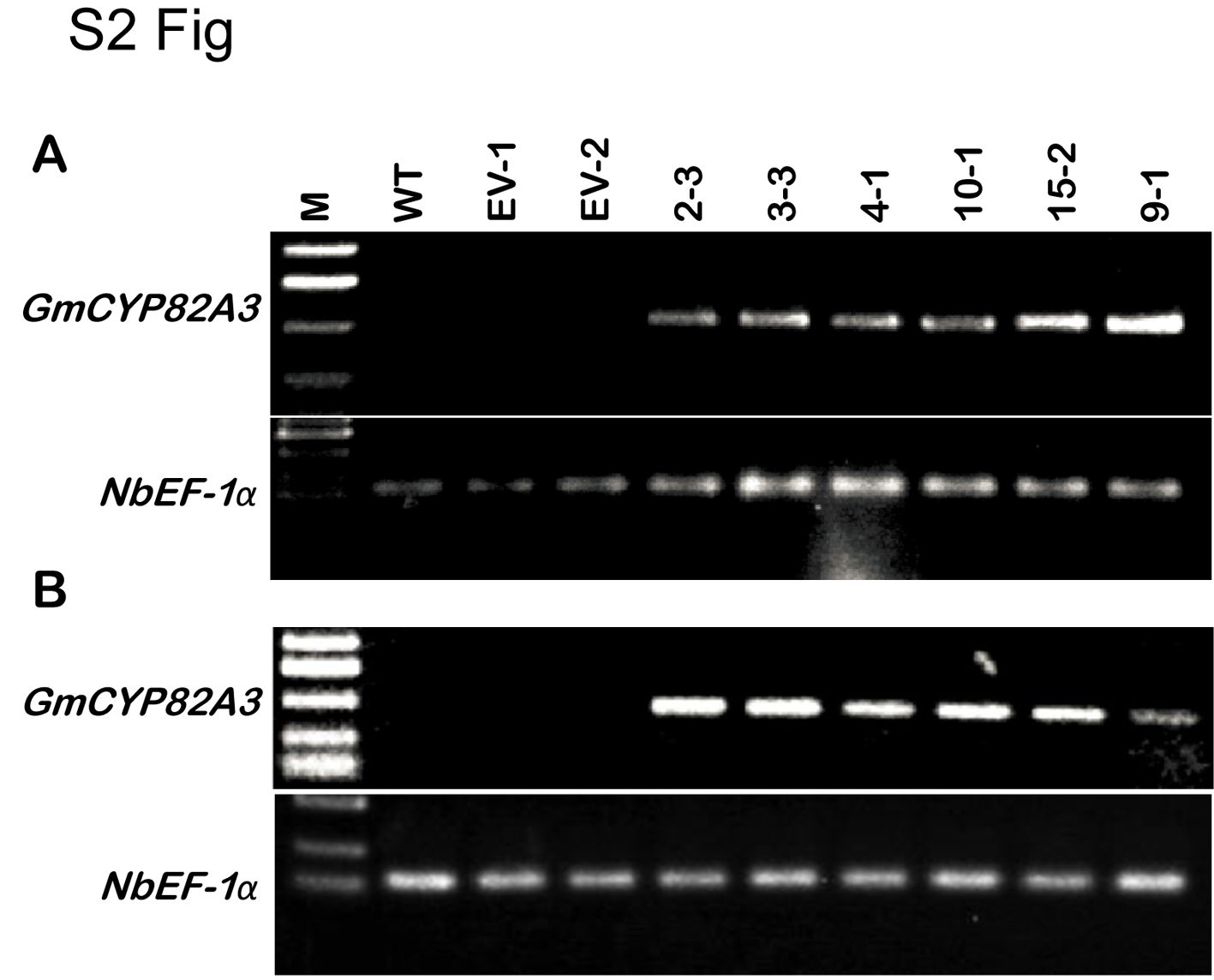

Supplement: S2 Fig — Electrophoresis pattern of PCR from genomic DNA (A) and cDNA (B) corresponding to GmCYP82A3 (upper panel) of wild-type (WT), empty vector (EV) transformants (EV-1, EV-2) and the six T2 transgenic lines expressing GmCYP82A3 (2–3, 3–3, 4–1, 10–1, 15–2, and 9–1). The NbEF1a (lower panel) was used as an internal control. M, DNA Marker DL2000 PLUS. (TIF) [file pone.0162253.s003.tif]

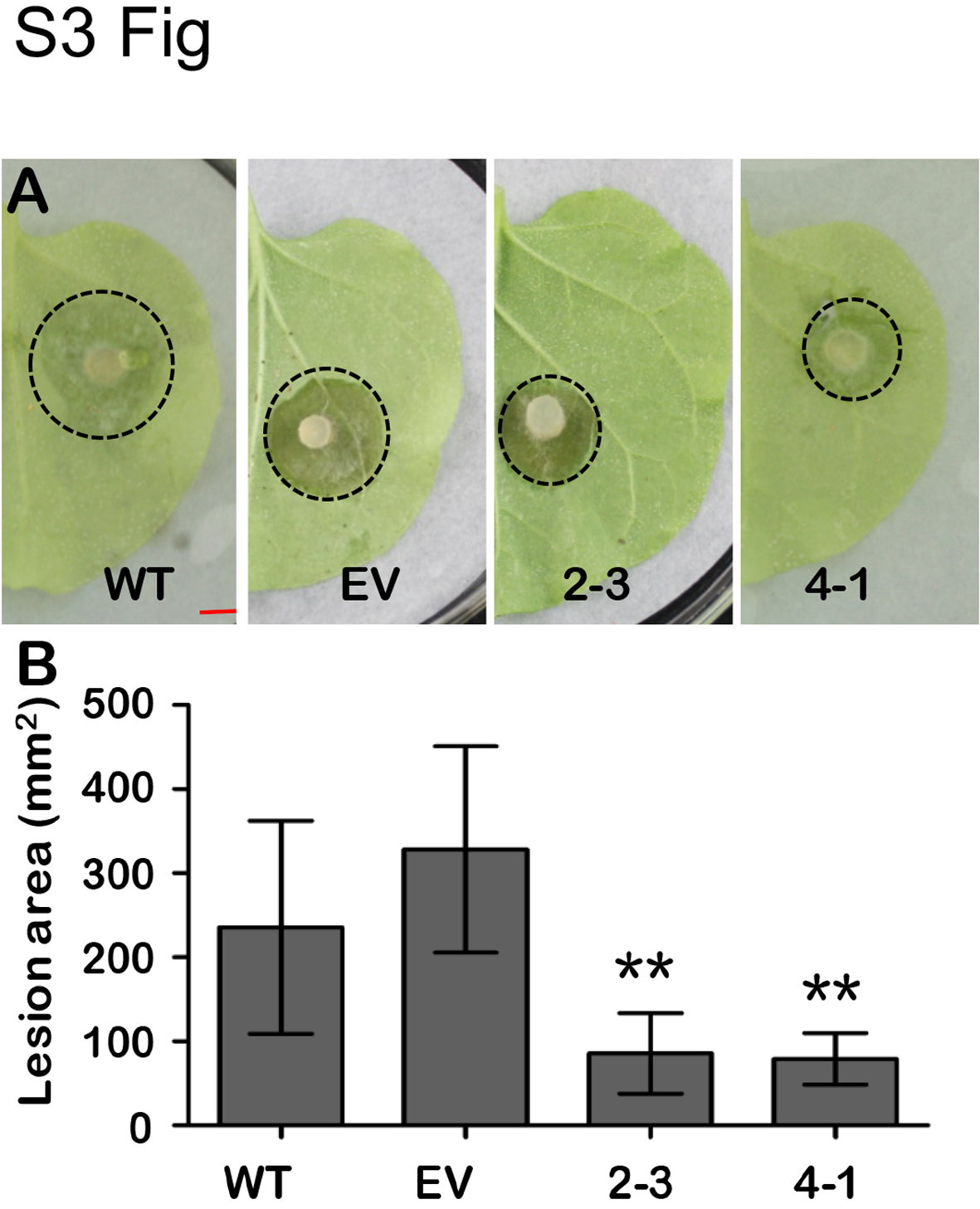

Supplement: S3 Fig — (A) Phenotypes of the N. benthamiana leaves from WT, EV and overexpression lines (2–3 and 4–1) inoculated with B. cinerea at 4 dpi. Bar = 5 mm. (B) Lesion area of inoculated leaves. Lesion diameters were measured at 4 dpi and then the lesion area was calculated. SD represented with the bars (Dunnett-t test: ** P<0.01). (TIF) [file pone.0162253.s004.tif]
